# An exploratory study on counterfactual thinking in amyotrophic lateral sclerosis

**DOI:** 10.3389/fpsyg.2023.1281976

**Published:** 2023-12-04

**Authors:** Barbara Poletti, Edoardo Nicolò Aiello, Sofia Tagini, Federica Solca, Silvia Torre, Eleonora Colombo, Alessio Maranzano, Ruggero Bonetti, Francesco Schevegher, Claudia Morelli, Alberto Doretti, Federico Verde, Sergio Barbieri, Francesca Mameli, Alberto Priori, Roberta Ferrucci, Vincenzo Silani, Paolo Cherubini, Gabriella Pravettoni, Nicola Ticozzi

**Affiliations:** ^1^Department of Neurology and Laboratory of Neuroscience, IRCCS Istituto Auxologico Italiano, Milano, Italy; ^2^Department of Oncology and Hemato-Oncology, University of Milan, Milano, Italy; ^3^“Rita Levi Montalcini” Department of Neurosciences, University of Turin, Torino, Italy; ^4^Neurology Residency Program, Università degli Studi di Milano, Milano, Italy; ^5^Department of Pathophysiology and Transplantation, “Dino Ferrari” Center, Università degli Studi di Milano, Milano, Italy; ^6^Fondazione IRCCS Ca' Granda Ospedale Maggiore Policlinico, Milano, Italy; ^7^Aldo Ravelli Center for Neurotechnology and Experimental Brain Therapeutics, Department of Health Sciences, International Medical School, University of Milan, Milano, Italy; ^8^ASST Santi Paolo e Carlo, San Paolo University Hospital, Milano, Italy; ^9^Department of Neural and Behavioral Sciences, University of Pavia, Pavia, Italy; ^10^Milan Center for Neuroscience, University of Milano-Bicocca, Milano, Italy; ^11^Applied Research Division for Cognitive and Psychological Science, IEO, European Institute of Oncology, IRCCS, Milano, Italy

**Keywords:** counterfactual thinking, cognition, amyotrophic lateral sclerosis, frontotemporal degeneration, neuropsychology, dementia

## Abstract

**Objectives:**

This study aimed at exploring (1) the motor and non-motor correlates of counterfactual thinking (CFT) abilities in non-demented amyotrophic lateral sclerosis (ALS) patients and (2) the ability of CFT measures to discriminate these patients from healthy controls (HCs) and patients with and without cognitive impairment.

**Methods:**

*N* = 110 ALS patients and *N* = 51 HCs were administered two CFT tasks, whose sum, resulting in a CFT Index (CFTI), was addressed as the outcome. Patients further underwent an in-depth cognitive, behavioral, and motor-functional evaluation. Correlational analyses were run to explore the correlates of the CFTI in patients. Logistic regressions were performed to test whether the CFTI could discriminate patients from HCs.

**Results:**

The CFTI was selectively associated (*p* ≤ 0.005) with fluency and memory subscales of the Edinburgh Cognitive and Behavioral ALS Screen (ECAS), but not with other variables. CFTI scores discriminated patients from HCs (*p* < 0.001) with high accuracy (82%), but not patients with a normal vs. defective performance on the ECAS-Total.

**Conclusion:**

CFT measures in non-demented ALS patients were associated with verbal fluency and memory functions, and they were also able to discriminate them from HCs.

## 1 Introduction

Counterfactual thinking (CFT) is defined as one's own ability to cognitively simulate alternatives to events that have occurred in order to regulate complex behaviors at both individual and social levels (Roese, 1997; Epstude and Roese, 2008). Counterfactual thoughts are believed to be “physiologically” triggered (“activation” stage of CFT) by affective states and/or situational occurrences that lead individuals to think (“content” stage of CFT) about how the outcomes of a given course of events could have been better—i.e., “upward” CFT or worse and “downward” CFT—had they acted differently (Roese, 1997; Epstude and Roese, 2008). In these terms, CFT processes are theorized to functionally allow humans to regulate future behaviors based on past events (Roese, 1997; Epstude and Roese, 2008).

As far as neural substrates are concerned, CFT abilities are believed to be subserved by widespread cortical-subcortical frontotemporal networks involved in both executive and episodic memory functions (De Brigard et al., 2013, 2015; Van Hoeck et al., 2013; De Brigard and Parikh, 2019).

CFT deficits have been documented in a variety of brain disorders of focal, developmental, degenerative, and demyelinating etiologies, as well as in psychiatric conditions (Tagini et al., 2021). With specific regard to neurodegenerative diseases, CFT deficits have been previously described in Parkinson's (McNamara et al., 2003) and Huntington's disease (Solca et al., 2015) patients only. Therein, CFT measures were found to be associated with tests tapping on both executive and social-cognitive functions.

However, no study to date has explored CFT abilities in patients with amyotrophic lateral sclerosis (ALS), up to 50% of whom present with cognitive deficits within the *spectrum* of frontotemporal degeneration (Strong et al., 2017). Nevertheless, given the likely link between CFT and both executive and social-cognitive functions (McNamara et al., 2003; Solca et al., 2015), which are acknowledged to negatively affect prognosis in this population by undermining decision-making and adherence within care settings (Huynh et al., 2020), the assessment of CFT abilities in ALS patients might convey relevant clinical entailments.

Given the above premises, the present study aimed at exploratively assessing (1) the motor and non-motor correlates of CFT abilities in a cohort of non-demented ALS patients, (2) the capability of CFT measures to discriminate patients from healthy controls (HCs), and (3) patients with vs. without cognitive impairment.

## 2 Methods

### 2.1 Participants

*N* = 110 consecutive, El Escorial-diagnosed ALS patients referred to IRCCS Istituto Auxologico Italiano, Milano, Italy, between 2021 and 2022 were consecutively recruited, along with *N* = 51 healthy controls (HCs) identified *via* the authors' acquaintances. No patient had a comorbid diagnosis of either behavioral variant-frontotemporal dementia or progressive non-fluent aphasia/semantic dementia. Both patients and HCs were free of (1) other neurological/psychiatric conditions, (2) severe/unstable general medical diseases, and (3) uncorrected vision/hearing deficits. This study was approved by the Ethics Committee of IRCCS Istituto Auxologico Italiano (I.D.: 2021_03_23_03); participants provided written informed consent.

### 2.2 Materials

Both patients and HCs were administered two CFT tasks, i.e., the Counterfactual Inference Test (CIT) (Hooker et al., 2000) and the Spontaneous Counterfactual Generation Test (SCGT) (Roese and Hur, 1997). The CIT requires the examinee to determine who, among two characters having experienced similar, but not identical, circumstances, is more likely to generate “if only”-like thoughts. The CIT is a 4-item, forced-choice task ranging from 0 to 4. By contrast, the SCGT first requires the examinee to recall a past life event and analyze it in detail for 3 min; he/she is then asked to propose alternative conclusions to such an occurrence. The outcome of the SCGT is represented by the number of alternative conclusions provided by the examinee; it is thus an open-range task. Both the CIT and the SCGT have been previously adapted for administration to Italian examinees (Zago et al., 2014; Solca et al., 2015).

Additionally, patients were assessed for global cognition [Edinburgh Cognitive and Behavioral ALS Screen, ECAS (Poletti et al., 2016)], executive [Frontal Assessment Battery, FAB (Appollonio et al., 2005; Aiello et al., 2023a)], social-cognitive functions [Reading the Mind in the Eyes Test, RMET (Aiello et al., 2022; Maddaluno et al., 2022); Emotion Attribution subtest of the Story-Based Empathy Task, SET-EA (Dodich et al., 2015; Aiello et al., 2023b)], behavior [State- and Trait-Anxiety Inventory-Form Y, STAI-Y1/-Y2 (Spielberger et al., 1971); Beck Depression Inventory, BDI (Beck et al., 1961); Beaumont Behavioral Inventory, BBI (Iazzolino et al., 2022); Dimensional Apathy Scale, DAS (Santangelo et al., 2017)], and motor-functional status [ALS Functional Rating Scale-Revised, ALSFRS-R (Cedarbaum et al., 1999)]. Progression rate (ΔFS) was computed as (48-ALSFRS-R score)/disease duration in months (Kimura et al., 2006), and disease staging was retrieved *via* King's (Roche et al., 2012) and Milano-Torino (MiToS) (Chiò et al., 2015) systems.

### 2.3 Statistics

Due to the limited ranges of both the CIT and the SCGT, a CFT index (CFTI) was computed as the sum of the two tasks and addressed as the outcome for the analyses, as the two tasks proved to be unrelated both in patients [*r*_(110)_ = 0.15; *p* = 0.122] and in HCs [*r*_(51)_ = −0.05; *p* = 0.709].

In patients, the association between age/education and sex and CFTI scores was assessed *via* Pearson's correlations and an independent-sample *t*-test, respectively, as indexed by both skewness and kurtosis values < |1| and |3|, respectively, as well as by the absence of visual abnormalities within their histogram and Q-Q plot.

By contrast, since the vast majority of remaining measures did not distribute normally according to the abovementioned criteria, non-parametric techniques were adopted to test associations of interest.

More specifically, three different sets of Bonferroni-corrected Spearman's correlations were run to test the association between the CFTI and (1) cognitive (i.e., ECAS-Language/-Fluency/-Executive/-Memory/-Visuospatial scores as well as FAB, RMET, and SET scores), (2) behavioral (i.e., STAI-Y1/-Y2, BDI, BBI, and DAS scores), and (3) motor-functional outcomes (i.e., disease duration in months, ALSFRS-R scores, and ΔFS).

Finally, two logistic regressions were performed in order to test the capability of the CFTI to (1) discriminate patients from HCs and, among the patient cohort, (2) discriminate cognitively impaired patients from cognitively unimpaired ones [i.e., those with a below- vs. above-cutoff ECAS-Total according to current, age- and education-stratified Italian norms (Poletti et al., 2016)]. Since patients and HCs were matched for education [*t*_(159)_ = 1; *p* = 0.321] but not for age [*t*_(159)_ = −6.01; *p* < 0.001] and sex [$\chi_{\left(1\right)}^{2}$ = 10.02; *p* = 0.002], the last two variables were covaried when running the first model. At variance, patients with a normal (*N* = 81) vs. defective performance (*N* = 29) on the ECAS-Total were matched for age [*t*_(108)_ = 0.28; *p* = 0.778], education [*t*_(40.31)_ = −1.42; *p* = 0.163], and sex [$\chi_{\left(1\right)}^{2}$ = 0.24; *p* = 0.621].

Analyses were run *via* jamovi 2.3 (https://www.jamovi.org/). Missing data were excluded pairwise.

## 3 Results

Table 1 summarizes the participants' background and clinical and neuropsychological measures. In patients, CFTI scores were unrelated to age [*r*_(110)_ = 0.08; *p* = 0.401], education [*r*_(110)_ = 0.10; *p* = 0.302], and sex [*t*_(108)_ = −0.84; *p* = 0.402].

**Table 1:** Participants' background and neuropsychological measures.

	**ALS**	**HCs**	***p***
***N***	110	51	-
**Sex (M/F)**	64/46	16/35	0.002^a^
**Age (y.)**	62.8 ± 10.3 (28–82)	52.1 ± 11.3 (36–75)	< 0.001^b^
**Education (y.)**	12.4 ± 3.9 (5–18)	13.1 ± 4.0 (5–19)	0.625^b^
**Disease duration (months)**	18.4 ± 18.6 (1–108)	-	-
**ALSFRS-R**	40.7 ± 5.3 (22–48)	-	-
**ΔFS**	0.7 ± 0.8 (0–5.2)	-	-
**NIV (%)**	0%	-	-
**PEG (%)**	1.0%	-	-
**King's (%)**			
Stage 1	41.9%	-	-
Stage 2	32.3%	-	-
Stage 3	20.4%	-	-
Stage 4	2.2%	-	-
**MiToS (%)**			
Stage 0	86.0%	-	-
Stage 1	14.0%	-	-
**ECAS**			
Total	102.2 ± 16.4 (34–127)	-	-
ALS-specific	75.6 ± 13.3 (25–96)	-	-
ALS Non-specific	26.6 ± 4.7 (9–33)	-	-
Language	24.5 ± 3.2 (15–28)	-	-
Fluency	17.3 ± 5.0 (0–24)	-	-
Executive functions	33.8 ± 7.6 (10–46)	-	-
Memory	15.2 ± 4 (1–21)	-	-
Visuospatial	11.4 ± 1.3 (5–12)	-	-
**FAB**	16.2 ± 1.9 (9–18)	-	-
**RMET**	22.2 ± 4.5 (10–32)	-	-
**SET-EA**	3.9 ± 1.6 (0–6)	-	-
**CIT**	2.0 ± 1.1 (0–4)	2.8 ± 1.3 (0–4)	-
**SCGT**	1.3 ± 1.0 (0–4)	2.2 ± 1.2 (0–6)	-
**CFTI**	3.2 ± 1.6 (0–7)	5.0 ± 1.7 (1–8)	-
**STAI-Y1**	53.7 ± 11.2 (23–87)	-	-
**STAI-Y2**	49.5 ± 8.9 (20–73)	-	-
**BDI**	12.7 ± 7.9 (0–37)	-	-
**BBI**	2.9 ± 2.8 (0–13)	-	-
**DAS**	22.4 ± 7.3 (3–40)	-	-

ALS, amyotrophic laterals sclerosis; ALSFRS-R, Amyotrophic Lateral Sclerosis Functional Rating Scale-Revised; ΔFS, progression rate; BBI, Beaumont Behavioral Inventory; BDI, Beck Depression Inventory; CFTI, Counterfactual Thinking Index; CIT, Counterfactual Inference Test; DAS, Dimensional Apathy Scale; ECAS, Edinburgh Cognitive and Behavioral ALS Screen; F, female; HCs, healthy controls; M, male; NIV, non-invasive ventilation; PEG, percutaneous endoscopic gastrostomy; SCGT, Spontaneous Counterfactual Generation Test; SET-EA, Story-Based Empathy Task-Emotion Attribution; STAI-Y1, State- and Trait-Anxiety Inventory-Form Y—State-Anxiety; STAI-Y2, State and Trait Anxiety Inventory-Form Y—Trait. ^a^χ^2^ statistics. ^b^*t* statistics.

At α_adjusted_ = 0.017, no associations were detected between CFTI scores and motor-functional outcomes (*p*s ≥ 0.104). Similarly, at α_adjusted_ = 0.01, the CFTI was unrelated to STAI-Y1, STAI-Y2, BDI, BBI, and DAS scores (*p*s ≥ 0.160). By contrast, at α_adjusted_ = 0.006, CFTI scores were associated with both ECAS-Fluency [*r*__*s*_(110)_ = 0.28; *p* = 0.003] and ECAS-Memory scores [*r*__*s*_(110)_ = 0.27; *p* = 0.005], while not with ECAS-Language/-Executive/-Visuospatial, RMET, SET-EA, and FAB scores (*p*s ≥ 0.074).

**Figure 1:**
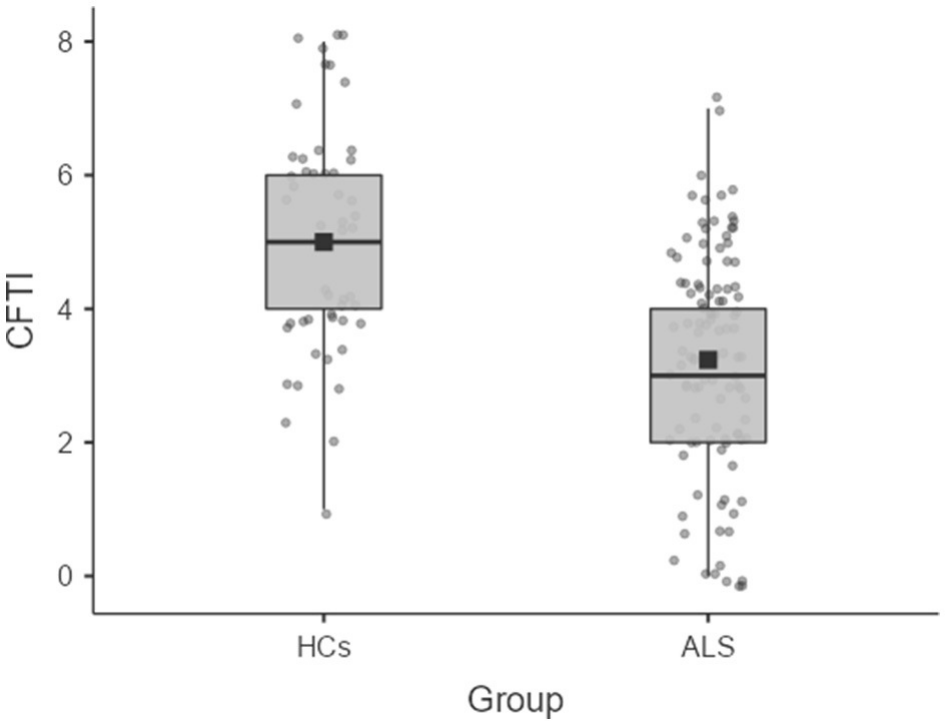
Box-plot showing the comparisons of the CFTI between ALS patients and HCs. ALS, amyotrophic laterals sclerosis; CFTI, counterfactual thinking index; HCs, healthy controls.

The CFTI was able to discriminate patients from HCs [*b* = −0.50, *z* = −3.80, *p* < 0.001; OR = 0.60, CI 95% (0.47, 0.78)] with high accuracy (82%) (Figure 1), but was unable to discriminate patients with vs. without a defective ECAS-Total score [*b* = 0.19, *z* = −1.43, *p* = 0.153; OR = 0.60, CI 95% (0.47, 0.78)].

## 4 Discussion

The present study, for the first time, explored the *status* of CFT abilities in non-demented ALS patients. CFT measures were herewith found to be selectively associated with verbal fluency and memory functions, while being independent of demographic, motor-functional, and behavioral features.

Such findings are, in the first place, likely to be accounted for by the nature of the tasks herewith adopted for measuring such a construct. Indeed, both the CIT and the SCGT require, albeit to different extents, to recall and generate verbal items—similarly to verbal fluency and verbal memory tasks included within the ECAS-Fluency and the ECAS-Memory, respectively. At the same time, they appear to align with the notion of executive and memory functions being related to CFT abilities (De Brigard and Parikh, 2019). However, in this respect, the non-negligible, inherent overlap between the current CFT measures and the tasks enclosed within the ECAS-Fluency/-Memory—both requiring the elicitation of reconstructive-recalling processes and relying on verbal responses—suggests that the associations herewith detected might be of a spurious nature. Hence, further investigations are needed in order to elucidate the cognitive underpinnings of CFT abilities in ALS.

Notably, the present results suggest that CFT abilities in this population are not accounted for by executive-attentive processes *per se*—this is in contrast with previous evidence reported in Parkinson's and Huntington's disease patients (McNamara et al., 2003; Solca et al., 2015). Such a discrepancy might be accounted for by the fact that Parkinson's and Huntington's disease are by default characterized by a primary neurodegeneration of networks subserving cognitive functions; by contrast, cognitive involvement in ALS is not ubiquitous since it appears only when extra-motor regions are involved.

As to the lack of associations between CFT measures and both motor-functional and behavioral features, it might be hypothesized that such findings are due to the fact that the patients herewith enrolled were in the early stages of the disease.

Interestingly, CFT measures were shown to be able to accurately discriminate non-demented ALS patients from HCs, suggesting that CFT abilities might be, at least to some extent, defective in this population. Nevertheless, the unavailability of Italian normative data for the CIT and the SCGT prevented estimating the actual prevalence of CFT deficits within the present cohort.

At the same time, the CFTI herewith proved to be unable to differentiate cognitively impaired from unimpaired patients. Hence, it might be postulated that the decrease in CFT abilities in this population is independent of the presence of overall cognitive impairment. In this respect, at least two putative explanations can be brought up. The first relates to the fact that the proportion of cognitive impairment within the present cohort was lower when compared to those commonly accepted within the relevant literature (i.e., up to 50%) (Murphy et al., 2016; Strong et al., 2017). This might have reduced the statistical power of the concerning comparison, thus confounding a potentially significant difference. As to the second, a measurement-related issue might be taken into account: indeed, cognition has been herewith assessed *via* a first-level test (i.e., the ECAS). It cannot be ruled out that a significant difference in CFT abilities might have been detected if function-/domain-specific tests had been employed for classifying patients' cognitive status. Thus, further investigations are needed that address a larger and more deeply phenotyped sample of patients in order to determine whether CFT measures could discriminate between ALS patients with and without cognitive dysfunctions. Relatedly, future studies should also include ALS patients with comorbid frontotemporal dementia.

Finally, a number of limitations of this study need to be highlighted. First, it is worth noting that the current investigation mostly embraced the “positive” polarity of CFT—i.e., its functional effects in terms of behavior regulation. However, it has been postulated that the excessive generation of counterfactuals might be linked to ruminative thoughts, which in turn might lead to anxiety and depressive symptoms (Epstude and Roese, 2008). Hence, while no associations were herewith detected between the CFTI and BDI/STAI-Y scores, it is advisable that future studies also focus on exploring whether excessive CFT might negatively contribute to ALS patients' psychological wellbeing. Second, it has to be borne in mind that the correlations herewith run for testing the association between CFT measures and cognitive scores have not been covaried for potentially relevant confounders, such as behavioral, psychopathological, and motor-functional features. At variance, the association between such confounders and the CFTI has been separately tested within different correlational sets. Although this was the aim of this explorative investigation, future studies are advisable that focus on exploring the determinants of CFT abilities in these patients by employing multivariate models. A third issue then lies in the fact that the patient cohort was much larger when compared to the HP group; this could have led, at least to some extent, to biases in the results of between-group comparisons, thus calling for future investigations to be adequately balanced in such terms.

Overall, while the present study is intended to be exploratory, it prompts further research aimed to determine whether CFT measures convey clinical entailments in non-demented ALS patients. Future studies are indeed advised to focus on assessing whether CFT abilities in this population are linked to decision-making and adherence within care settings, especially within the context of advanced care planning.

In conclusion, CFT abilities in non-demented ALS patients are associated with verbal fluency and memory functions; moreover, CFT measures are able to discriminate such patients from HCs.

## Data availability statement

Raw data have been stored on an online repository (https://zenodo.org/records/10159681) and can be made available upon reasonable request to the corresponding author.

## Ethics statement

The studies involving humans were approved by Ethics Committee of IRCCS Istituto Auxologico Italiano (I.D.: 2021_03_23_03). The studies were conducted in accordance with the local legislation and institutional requirements. The participants provided their written informed consent to participate in this study.

## Author contributions

BP: Conceptualization, Investigation, Project administration, Resources, Supervision, Validation, Writing – original draft, Writing – review & editing. EA: Formal analysis, Methodology, Software, Writing – original draft, Writing – review & editing. STa: Conceptualization, Investigation, Methodology, Writing – review & editing. FSo: Conceptualization, Investigation, Methodology, Writing – original draft, Writing – review & editing. STo: Data curation, Investigation, Writing – review & editing. EC: Data curation, Investigation, Writing – review & editing. AM: Data curation, Investigation, Writing – review & editing. RB: Data curation, Investigation, Writing – review & editing. FSc: Data curation, Investigation, Writing – review & editing. CM: Data curation, Investigation, Supervision, Writing – review & editing. AD: Data curation, Investigation, Supervision, Writing – review & editing. FV: Data curation, Investigation, Supervision, Writing – original draft, Writing – review & editing. SB: Resources, Supervision, Writing – review & editing. FM: Supervision, Writing – review & editing. AP: Supervision, Writing – review & editing. RF: Supervision, Validation, Writing – review & editing. VS: Funding acquisition, Resources, Supervision, Writing – review & editing. PC: Supervision, Writing – review & editing. GP: Supervision, Validation, Writing – review & editing. NT: Funding acquisition, Resources, Supervision, Writing – review & editing.
